# Cationic Carbon Nanoparticles Induce Inflammasome-Dependent Pyroptosis in Macrophages *via* Lysosomal Dysfunction

**DOI:** 10.3389/ftox.2022.925399

**Published:** 2022-07-19

**Authors:** Yasmin Arezki, Mickaël Rapp, Luc Lebeau, Carole Ronzani, Françoise Pons

**Affiliations:** Laboratoire de Conception et Application de Molécules Bioactives, UMR 7199, CNRS-Université de Strasbourg, Faculté de Pharmacie, Illkirch, France

**Keywords:** carbon dot, pyroptosis, caspase-1, inflammasome, lysosome, cathepsin B, macrophages

## Abstract

Carbon nanomaterials, including carbon dots (CDs), form a growing family of engineered nanoparticles (NPs) with widespread applications. As the rapid expansion of nanotechnologies raises safety concerns, interaction of NPs with the immune system is receiving a lot of attention. Recent studies have reported that engineered NPs may induce macrophage death by pyroptosis. Therefore, this study investigated whether cationic CDs induce pyroptosis in human macrophages and assessed the role of inflammasome and lysosome in this process. Cationic CDs were synthetized by microwave-assisted pyrolysis of citric acid and high molecular weight branched polyethyleneimine. The NPs evoked a dose-dependent viability loss in THP-1-derived macrophages. A cell leakage, an increase in IL-1β secretion and an activation of caspase-1 were also observed in response to the NPs. Inhibition of caspase-1 decreased CD-induced cell leakage and IL-1β secretion, while restoring cell viability. Besides, CDs triggered swelling and loss of integrity of lysosome, and inhibition of the lysosomal enzyme cathepsin B decreased CD-induced IL-1β secretion. Thus, our data provide evidence that cationic CDs induce inflammasome-dependent pyroptosis in macrophages *via* lysosomal dysfunction.

## Introduction

In the last decades, many kinds of nanoparticles (NPs) with different chemical composition (carbon, metal, silica, lipid, polymer) have been engineered for various purposes, including drug delivery or biomedical imaging (Rudramurthy and Swamy, 2018). As the rapid expansion of nanotechnologies raises safety concerns, interaction of engineered nanomaterials with the immune system is receiving a lot of attention (Dobrovolskaia et al., 2016; Boraschi et al., 2017; Pallardy et al., 2017). Indeed, when entering the body, NPs can be recognized as foreign material and sensed as danger signals by the immune system, and particularly macrophages. Macrophages are one of the first lines of the host defense against a large variety of external stimuli and therefore essential effectors of the innate immune system (Laskin et al., 2011). As professional phagocytes, macrophages clear the body of cell debris, dead cells, viruses and bacteria. They secrete also a multitude of mediators including reactive oxidative species (ROS), proteases, cytokines and growth factors. These mediators trigger an acute inflammatory response following body injury or infection and regulate the host defense. As part of antigen-presenting cells, macrophages contribute also to adaptative immunity. A number of studies have shown that macrophages can recognize and internalize various kinds of NPs (Nakayama, 2018). This may contribute to NP clearance, but also to their immunotoxicity. Indeed, macrophages produce ROS and secrete cytokines following exposure to NPs (Bussy et al., 2012; de Luna et al., 2016; Brzicova et al., 2019; Casset et al., 2019). NPs also trigger NLRP3 inflammasome activation, lysosomal dysfunction, and cell death in macrophages (Hornung et al., 2008; Tahara et al., 2012; Xu et al., 2015; Tsugita et al., 2017; Ronzani et al., 2019). As a consequence, macrophages have been shown to play an important role in immune-mediated disorders caused by NPs *in vivo*, such as acute lung inflammation or fibrosis (Shvedova et al., 2005; Ronzani et al., 2012).

Carbonaceous nanomaterials including nanodiamonds, fullerenes, graphene, carbon nanotubes and carbon dots (CDs) form a growing family of materials with widespread applications (Cha et al., 2013). Discovered almost 2 decades ago, CDs are quasi-spherical particles with unique properties including, very small size (few nanometers), excellent water solubility, tunable intrinsic fluorescence, and resistance to photobleaching (Xu et al., 2004). Furthermore, they can be easily synthesized through a range of methods starting from cost effective materials, and their functionalization can be straightforwardly achieved, making them invaluable multifunctional platforms for designing sophisticated devices with huge potential for industrial applications (Himaja et al., 2015). Beside optoelectronics, photovoltaics, energy storage or wastewater treatment, CDs are currently developed for applications in nanomedicine including drug or gene delivery, biomedical imaging and theranostics (Pierrat et al., 2015; Claudel et al., 2019; Du et al., 2019; Ghosal and Ghosh, 2019). Although carbon is not considered as a toxic element, carbon-based nanomaterials have raised some safety concerns, particularly towards immune cells (Yuan et al., 2019). Concerning CDs, we and others reported internalization of the NPs by macrophages, associated with oxidative stress, cytokine secretion, and viability loss (Lategan et al., 2018; Ronzani et al., 2019). However, these effects vary depending on CD physicochemical characteristics, especially surface charge. Using a large library of NPs, we showed that cationic CDs, and particularly cationic NPs with a marked surface charge density induce macrophage viability loss, whereas anionic CDs exhibit no effect (Fan et al., 2019; Weiss et al., 2021b). Cationic CDs with a marked surface charge density trigger as well lung inflammation in mice upon airway administration, in contrast to anionic ones (Weiss et al., 2021b). The putative AOP leading to this pathological response involves uptake of the NPs by macrophages as molecular initiating event (Weiss et al., 2021a).

Pyroptosis is a lytic, pro-inflammatory form of programmed cell death that was first described in 1992 (Cookson and Brennan, 2001). It is mainly observed in macrophages, particularly after bacterial infection, and is characterized by loss of membrane integrity leading to the release of the cellular content, including damage-associated molecular patterns (DAMPs) and inflammatory cytokines such as IL-1β. Pyroptosis proceeds through two main pathways: 1-the canonical inflammasome pathway which is dependent on inflammasome and caspase-1 activation, and 2-the noncanonical pathway which is mediated by caspase-4/5/11 (Hachim et al., 2020). Recent studies have reported that engineered NPs may induce cell death by pyroptosis in murine or human hepatocytes or macrophages in an inflammasome-dependent manner (Reisetter et al., 2011; Lu et al., 2016; Mirshafiee et al., 2018; Zhang et al., 2018; Liang et al., 2020; Wang et al., 2020). Besides, some evidences suggest that lysosomal damage and cathepsins are implicated in pyroptosis under certain conditions (Wang et al., 2018). In the present study, we thus investigated whether cationic CDs exhibiting a high surface charge density trigger pyroptosis in macrophages and assessed the role of lysosome in this process. This work was carried out on phorbol 12-myristate 13-acetate (PMA)-differentiated THP-1 cells that represent a suitable model for studying macrophage functions *in vitro* (Chanput et al., 2014).

## Materials and Methods

### Preparation and Characterization of CDs

The CDs investigated herein were produced and characterized according to previously reported protocols (Fan et al., 2019; Ronzani et al., 2019; Weiss et al., 2021b). Briefly, for CD synthesis, citric acid (2.0 g) and branched polyethyleneimine 25,000 Da (bPEI 25 k, 8.0 g) were introduced in a 400 ml beaker and dissolved in ultrapure water (70 ml). The resulting clear mixture was stirred at 230–250°C until a thick dark brown sirup was obtained. The reaction mixture was cooled to rt, diluted with ultrapure water (35 ml), and neutralized with HCl 12 N. The solution obtained was then extensively dialyzed in a sealed 14 kDa MWCO bag against HCl 0.1 N (96 h, with frequent replacement of the dialysis medium), and against ultrapure water (24 h). The content of the dialysis bag was than filtered through a 0.22 μm PES membrane (Millex) and freeze-dried at −50°C for 24–36 h, to yield a brown hygroscopic fluffy powder (2.6 g). CD characterization was carried out on fresh CD suspensions (1.0 mg/ml) prepared in 1.5 mM NaCl pH 7.4. CD hydrodynamic diameter and ζ-potential were determined by dynamic light scattering (DLS) and electrophoretic light scattering (ELS) using a Zetasizer NanoZS apparatus (Malvern Instruments). Measurements were carried out in triplicate at 25°C and data were expressed as mean (±SD). The surface charge density of CDs was determined by polyelectrolyte titration, monitoring ζ-potential variation of CD suspension, along spiking with a solution of poly (acrylic acid) (PAA, MW ± 1,800 Da, NaCl 1.5 mM pH 7.4), as previously reported (Weiss et al., 2021b). The results were expressed in μmol/mg. Optical properties of CDs were determined by recording UV-visible and fluorescence spectra on CD preparations, using a multimode reader (Varioskan Lux, Thermo Fisher Scientific, France).

### Cell Culture

THP-1 cells (TIB-202™, ATCC) were grown in RPMI-1640 culture medium containing L-glutamine (2 mM), 2-mercaptoethanol (0.05 mM), penicillin (100 UI/mL), streptomycin (100 μg/ml), and heat inactivated fetal bovine serum (10%) at 37°C in a 5% CO_2_ humidified chamber (all culture reagents from GIBCO).

### Cell Exposure to CDs and Pharmacological Inhibitors

Cells were seeded in 96- or 24-well culture plates at a density of 1 × 10^5^ or 5 × 10^5^ cells/well, respectively and differentiated into macrophages by adding 10 ng/ml phorbol 12-myristate 13-acetate (PMA, Sigma) to culture medium overnight. Upon differentiation into macrophages, the cells become adherent. Differentiated cells were then washed with phosphate buffered saline (PBS) and incubated with freshly prepared CD solutions (3–100 μg/ml in complete culture medium) or with culture medium alone (controls) for 4 h (CD cell uptake, cell pyroptosis or cathepsin B activity) or 24 h (cell viability or necrosis, IL-1β release, lysosome swelling or integrity). In some experiments, cells were incubated with a caspase-1 (YVAD-CHO, 50 μM, Merck) or cathepsin B (CA-074Me, 10 μM, Enzo life) inhibitor for 1h30 at 37°C prior to CD exposure. All cell responses were analyzed at the end of the CD incubation period.

### Assessment of CD Cell Uptake

Fluorescence activated cell sorting (FACS) was used to assess CD uptake by macrophages thanks to the optical properties of the NPs, as previously described (Weiss et al., 2021b). Briefly, after exposure to CDs (25 μg/ml) for 4 h, the culture medium was discarded and the cells were rinsed twice with PBS and harvested by trypsin treatment. Cell suspensions were then analyzed with a LSRFortessa X 20^TM^ flow cytometer, by collecting sample fluorescence (20,000 events/sample) using a BV510 (violet laser) channel. CD uptake was quantified by determining changes in the mean of the fluorescence intensity (MFI) of CD-treated cells compared to untreated cells. Results were expressed as the ratio of the MFI of CD-treated cells to the MFI of untreated cells.

### Cell Viability

Cell viability was assessed by the MTT assay. Cells treated with CDs for 24 h were carefully washed with PBS before addition of MTT (100 μL, 1.0 mg/ml in complete culture medium, Sigma). After a 1-h incubation at 37°C, culture medium was removed, cells were lysed with dimethyl sulfoxide (100 μL), and absorbance of the resulting samples was read at 570 nm with a correction at 690 nm (Varioskan Lux multimode reader, Thermo Scientific). Cell viability was expressed as the percentage of the absorbance of CD-treated cells relative to the absorbance of control cells.

### Plasma Membrane Integrity

Plasma membrane integrity was assessed by measuring the release of lactate deshydrogenase (LDH) in the culture supernatants of cells exposed to CDs for 24 h, using the Cytotoxicity Detection Kit Plus (Roche Applied Science). LDH activity was measured according to the manufacturer’s instructions and expressed as the fold change in absorbance measured in the supernatants of CD-exposed cells relative to the absorbance measured in the supernatants of non-exposed cells (control).

### Caspase-1 Activity and Pyroptosis

Caspase-1 activity and pyroptosis were determined by flow cytometry using the FAM-FLICA^®^ assay (Biorbyt) and the low molecular weight DNA dye, propidium iodide (BD Biosciences). After exposure to CDs (25 and 100 μg/ml) for 4 h, the cells were washed with PBS, detached from the culture plate by trypsin treatment and centrifuged. Cells were then washed with culture medium, and resuspended in serum-free culture medium (290 μL) containing 10 μL of FAM-FLICA^®^ reagent (diluted one to five in PBS). After a 1-h incubation, each sample was washed with the assay kit buffer before flow cytometry analysis. For pyroptosis assessment, propidium iodide (1/100 dilution) was added to each sample before flow cytometry analysis. The sample fluorescence (20,000 events/sample) was measured with a LSRFortessa X-20™ cytometer (BD Biosciences) driven by the FACSDiva™ software (BD Biosciences), using the FITC (λ_ex_ 488 nm, λ_em_ 530 nm, FAM-FLICA^®^) and PE Texas Red (λ_ex_ 561 nm, λ_em_ 610 nm, propidium iodide) channels. Caspase-1 activity was expressed as change in FITC mean fluorescence intensity (MFI) of the cells exposed to CDs compared to control cells. Pyroptosis was expressed as the percentage of double-positive cells.

### Lysosomal Swelling

Lysosomal swelling was evaluated by flow cytometry using the lysosomal marker LysoTracker^®^ Red DND-99 (Molecular Probes). After a 24-h exposure to CDs (25 μg/ml), the cells were washed with PBS and incubated with 50 nM LysoTracker in serum-free culture medium for 30 min at 37°C. At the end of the incubation, the cells were washed with PBS, detached from the culture plates by trypsin treatment, centrifuged, and resuspended in serum-free culture medium (500 μL). The sample fluorescence (20,000 events/sample) was analyzed with excitation at 561 nm and signal detection at 586 nm (PE channel, yellow-green laser). Lysosomal swelling was expressed as change in MFI of the cells exposed to CDs compared to control cells.

### Lysosomal Integrity

The neutral red assay was used to assess changes in lysosomal integrity in response to CDs. Cell treated with CDs (3–100 μg/ml) for 24 h were carefully washed with PBS before addition of neutral red (200 μL of a 100 μg/ml solution in complete culture medium, Sigma). After a 3-h incubation period at 37°C, the cell supernatant was removed and the cells were washed with PBS, before addition of a neutral red extraction solution (200 μL of 1:1 water-ethanol solution containing 1% acetic acid) for 20 min. Absorbance of the resulting samples was measure at 540 nm (Varioskan Lux multimode reader, Thermo Scientific). Results were expressed as the percentage of the absorbance of CD-treated cells relative to the absorbance of control cells.

### Cathepsin B Activity

The activity of cathepsin B was quantified by fluorimetry using the Magic Red^®^ assay (CliniSciences). Cells were treated with CDs (25 and 100 μg/ml) for 4 h, washed with PBS and incubated with the cathepsin B substrate for 1 h at 37°C, according to the manufacturer’s instructions. At the end of the incubation period, the cells were washed with PBS, and the sample fluorescence was measured at 590 nm, with excitation at 540 nm (Varioskan Lux multimode reader, Thermo Scientific). The results were expressed as the ratio of fluorescence intensity measured in cells exposed to CDs compared to intensity measured in control cells.

### IL-1β Assay

Interleukin-1β (IL-1β) was quantified in the supernatant of cells incubated with CDs (3–100 μg/ml) for 24 h by ELISA (Biotechne, France). The assay was conducted according to the manufacturer’s instructions. Absorbance was read at 450 nm with a correction at 570 nm (Varioskan Lux multimode reader, Thermo Scientific). Cytokine concentrations were expressed in pg/mL.

### Statistical Analysis of the Data

Data were expressed as mean ± SEM and plotted as bar charts. Concentration-response curves (cell viability and lysosomal integrity) were obtained after logarithmic transformation of the data and fit with the Hill’s equation, whose slope was used to calculate a half maximal effective concentration (EC50). Statistical differences between groups were determined by a *t*-test or one- or two-way analysis of variance (ANOVA) followed by a Dunnett’s or Sidak’s test, using the GraphPad Prism 6.0 software. Data were considered as significantly different when *p* value was less than 0.05.

## Results

### CD Characteristics, Cell Uptake and Toxicity Towards Macrophages

**FIGURE 1:**
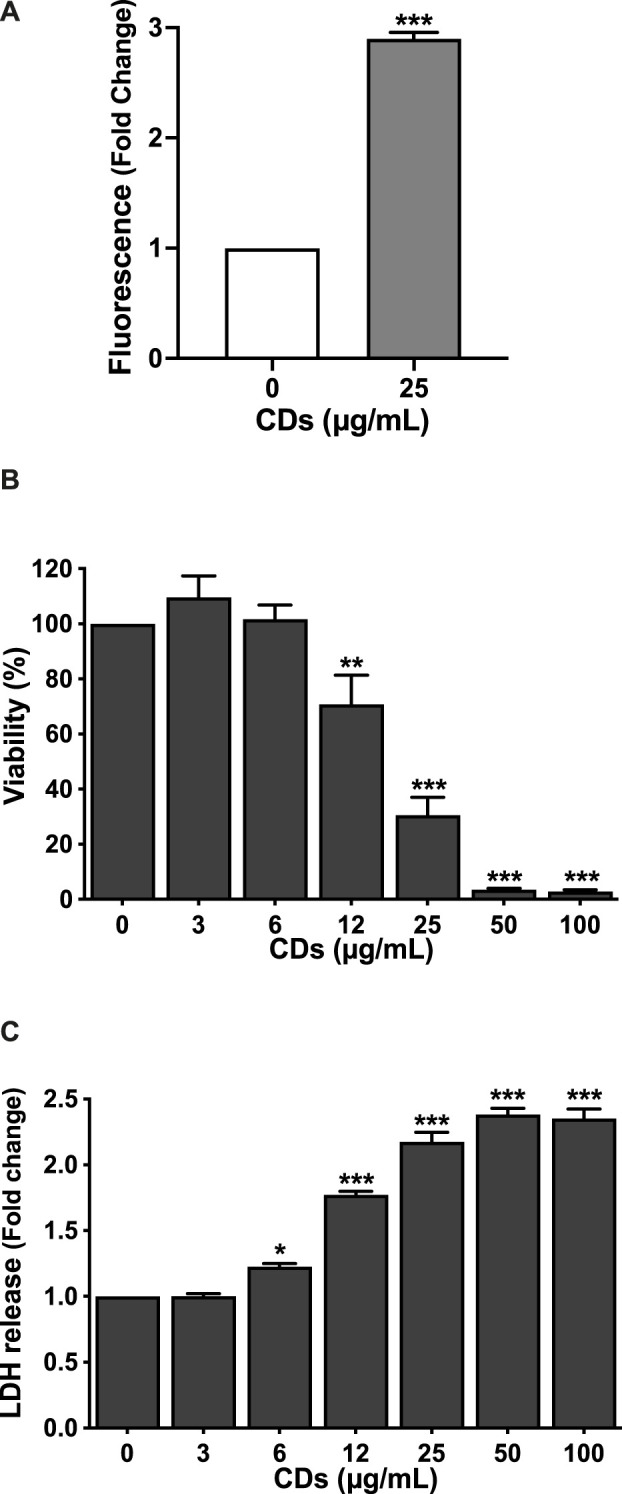
CD uptake and toxicity towards macrophages. **(A)** CD uptake by macrophages as assessed by FACS in cells exposed to the NPs (25 μg/ml) for 4 h. Results are expressed as fold change in fluorescence intensity when compared to control cells exposed to culture medium alone. They are means ± SEM of *n* = 3 experiments. Statistical differences between groups were determined by Student’s t-test. ***p < 0.001. **(B,C)** Cytotoxicity of CDs towards macrophages. Cells were exposed to increasing concentrations of CDs (3–100 μg/ml), or to culture medium alone (control) for 24 h, before cell viability (B, MTT assay) and cell membrane leakage (C, LDH assay) assessment. Results are expressed as percent (viability) or fold change (LDH release) relative to the control. They are the mean ± SEM of *n* = 3–4 experiments. Statistical differences when compared to control were determined by ANOVA followed by the Dunnett’s test. *p < 0.05, **p < 0.01 and ***p < 0.001.

The characteristics of CDs are presented in Table 1. The CDs had a mean hydrodynamic diameter of 9.9 ± 0.5 nm and their ζ-potential and charge density was of +32.4 ± 1.3 mV and 5.5 μmol/mg, respectively. As well, their maximum fluorescence excitation and emission wavelengths were at 350 and 460, respectively. Thus, the CDs used in herein were of small size, had a cationic charge with a high charge density and exhibited intrinsic fluorescence properties. These expected characteristics are in agreement with previous reports (Weiss et al., 2021b). As toxicity of NPs generally involves their cell entry, we first investigated uptake of CDs by macrophages using FACS, according to previous studies (Ronzani et al., 2019; Weiss et al., 2021b). As shown on Figure 1A, a significant CD-associated fluorescence signal (*p* < 0.001) was observed in macrophages exposed to the NPs (25 μg/ml) for 4 h, confirming CD internalization by the cells. To assess the cytotoxicity of CDs towards macrophages, cells were exposed to increasing concentrations of the NPs for 24 h, and cell viability was assessed by the MTT assay. CD concentrations ranging from 3 to 100 μg/ml were chosen according to previous studies (Fan et al., 2019; Ronzani et al., 2019). Figure 1B shows that CDs induce a concentration-dependent viability loss with a EC50 of 17.82 μg/ml (CI95 15.04–21.10 μg/ml). Viability loss was statistically significant at CD concentrations of 12 (*p* < 0.01), 25 (*p* < 0.001), 50 μg/ml (*p* < 0.001) and 100 μg/ml (*p* < 0.001), and reached c. a. 95% at 50 and 100 μg/ml. To further characterize the cell death caused by CDs, macrophage membrane integrity was assessed by measuring cell leakage with the LDH assay. As shown on Figure 1C dose-dependent cell leakage was observed in response to CDs. This leakage increased thus in parallel with the decrease in cell viability and was statistically significant at 12 (*p* < 0.001), 25 (*p* < 0.001), 50 μg/ml (*p* < 0.001) and 100 μg/ml (*p* < 0.001). Thus, the CDs investigated herein induce macrophage death.

**TABLE 1:** Physicochemical characteristics of the CDs.

	
Structure	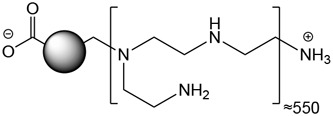
ζ-potential [mV]	+32.4 ± 1.3
Surface charge density Q_ek_ [µmol/mg]	5.5
Hydrodynamic diameter [nm]	9.9 ± 0.5
Optical properties λ_max_/λ_ex_/λ_em_ [nm]	^a^/350/460

a Monotone and decreasing UV-vis, absorption between 250 and 800 nm.

### CDs Induce Macrophage Pyroptosis

**FIGURE 2:**
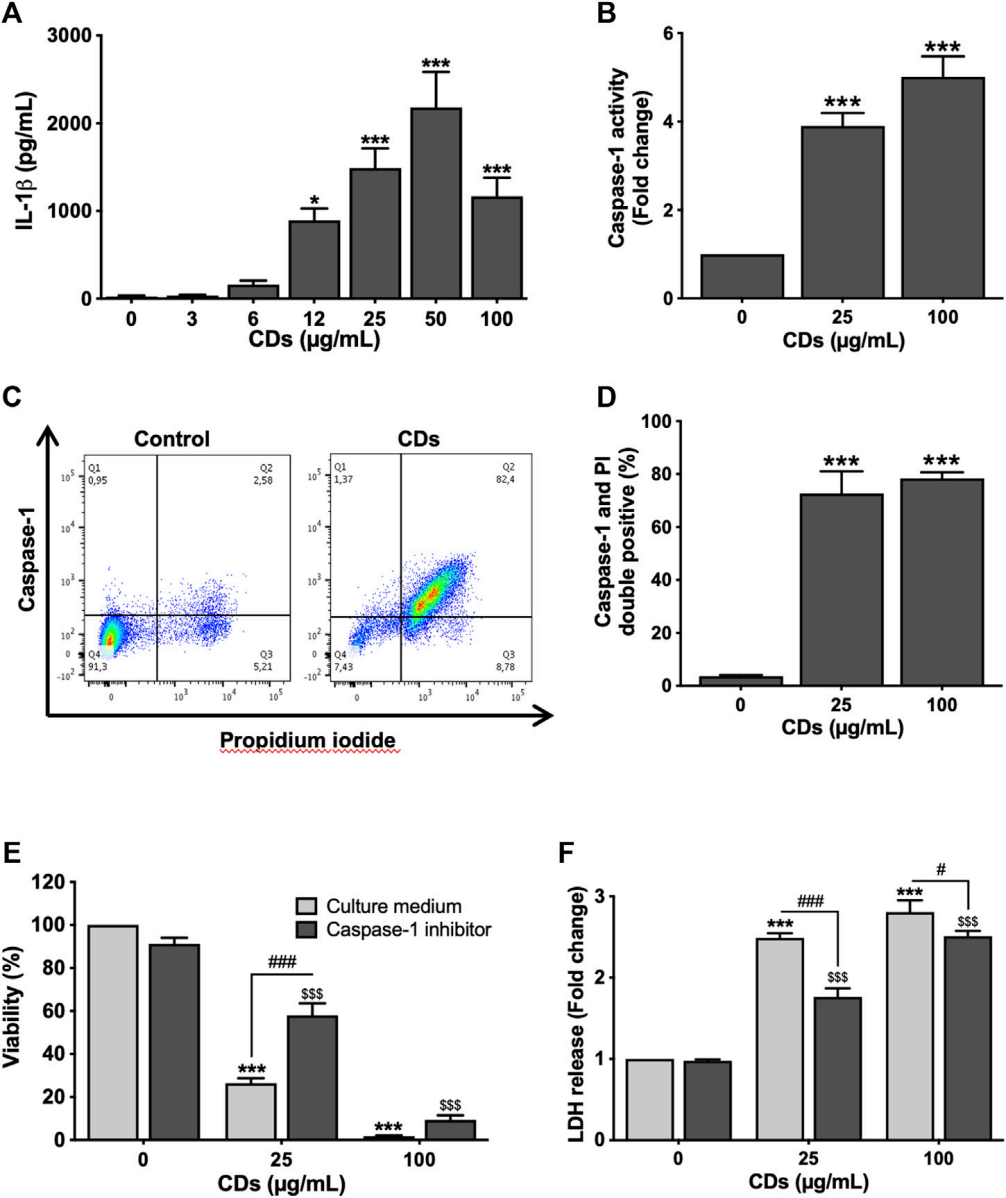
CDs induce macrophage pyroptosis. **(A,B)** Inflammasome activation. Macrophages were exposed to CDs (A: 3–100 μg/ml; B: 25 or 100 μg/ml) or culture medium alone (control) for 24 and 4 h before IL-1β secretion (A, ELISA) and caspase-1 activity (B, FACS) measurement, respectively. Results are expressed as absolute value (IL-1β secretion) or fold change relative to the control (caspase-1 activity). They are the mean ± SEM of *n* = 3–5 experiments. Statistical differences when compared to control cells were determined by ANOVA followed by the Dunnett’s test. *p < 0.05 and ***p < 0.001. **(C,D)** Macrophage death by pyroptosis. Cells were exposed to CDs (25 and 100 μg/ml CDs) or culture medium alone (control) for 4 h before pyroptosis assessment by flow cytometry. **(C)** Representative flow cytometry chart showing caspase-1 activity as a function of propidium iodide fluorescence in control and cells treated with 25 μg/ml CDs. **(D)** Pyroptosis induced by 25 and 100 μg/ml CDs, expressed as percent of double positive cells. Data are the mean ± SEM of *n* = 3 experiments. Statistical difference when compared to control was determined by ANOVA followed by the Dunnett’s test. ***p < 0.001. **(E,F)** Effect of caspase-1 inhibition on CD-induced macrophage death. Cells were incubated with the caspase-1 inhibitor YVAD-CHO (50 μM) for 1.5 h and then exposed to CDs (25 or 100 μg/ml) or culture medium alone (control) for 24 h before viability and membrane leakage assessment. Results are expressed as percent (MTT test) or fold change (LDH leakage) relative to the control. They are the mean ± SEM of *n* = 3–6 experiments. Statistical differences as determined by two-ways ANOVA followed by the Sidak’s test. ***p < 0.001, when compared to cells without inhibitor pre-treatment and CD exposure. ^$$$^ *p* < 0.001, when compared to cells with inhibitor pre-treatment and without CD exposure. ^#^ *p* < 0.05 and ^###^ *p* < 0.001, when compared to cells without inhibitor pre-treatment and with CD exposure.

To investigate whether CDs trigger macrophage cell death by pyroptosis, we first assessed inflammasome activation in response to the NPs by measuring the two main markers of this cellular pathway, IL-1β release and caspase-1 activation. Indeed, inflammasome plays a central role in the canonical pathways of pyroptosis through caspase-1 activation leading to IL-1β maturation and cell membrane leakage due to pore formation (Hachim et al., 2020). To do so, macrophages were exposed to the NPs for 4 (caspase-1) or 24 h (IL-1β release). As shown on Figure 2A, CDs (3–100 μg/ml) induced a concentration-dependent release of IL-1β, that was statistically significant at CD concentrations of 12 (*p* < 0.05), 25 (*p* < 0.001), 50 μg/ml (*p* < 0.001) and 100 μg/ml (*p* < 0.001). As well, CDs (25 and 100 μg/ml) triggered an increase in caspase-1 activity (3.9-fold, *p* < 0.001 and 5.0-fold, *p* < 0.001, respectively), as assessed by flow cytometry using the FAM-FLICA assay (Figure 2B). To provide further evidence of macrophage pyroptosis in response to CDs, cells exposed to the NPs (25 and 100 μg/ml) for 4 h were double labelled with the FAM-FLICA reagent and propidium iodide, to simultaneously measure caspase-1 activity and membrane integrity loss. Then, double positive cells were identified as pyroptotic cells by flow cytometry, as shown for the dose of 25 μg/ml CDs on Figure 2C. Seventy-two and seventy-eight percent of macrophages exposed to 25 and 100 μg/ml CDs respectively, were double positive, compared to four percent in control culture (*p* < 0.001 for both CD doses), suggesting macrophage death by pyroptosis (Figure 2D). To confirm this hypothesis, we assessed the effect of caspase-1 inhibition on macrophage viability and membrane integrity loss induced by CDs (25 and 100 μg/ml), using the YVAD-CHO inhibitor. As shown on Figure 2, caspase-1 inhibition partially restored cell viability (Figure 2E, *p* < 0.001 for 25 μg/ml) and reduced membrane leakage, as evidenced by LDH release (Figure 2F, *p* < 0.001 for 25 μg/ml and *p* < 0.05 for 100 μg/ml) in macrophages exposed to CDs.

### Lysosome Mediates CD-Induced Macrophage Pyroptosis

**FIGURE 3:**
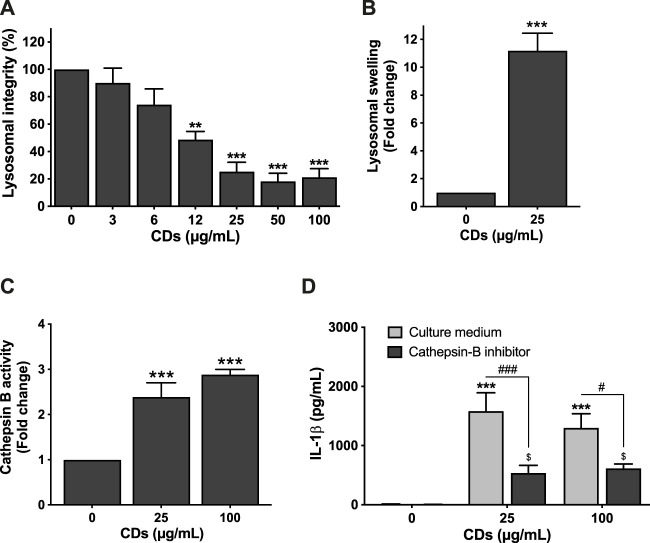
Lysosome mediates CD-induced macrophage pyroptosis. **(A,B)** CDs induce lysosomal dysfunction. Cells were exposed to CDs (A: 3–100 μg/ml; B: 25 μg/ml) or culture medium alone (control) for 24 h before assessment of lysosomal integrity (A, neutral red assay) and swelling (B, LysoTracker^®^ Red DND-99 staining). Results are expressed as fold change (lysosomal swelling) or percent (lysosomal integrity) relative to the control. They are the mean ± SEM of *n* = 3-6 experiments. Statistical differences when compared to control cells were determined by ANOVA followed by the Dunnett’s test **(A)** or by the *t*-test **(B)**. **p < 0.01 and ***p < 0.001. **(C)** Cathepsin B activity after macrophage exposure to CDs. Cells were exposed to CDs (25 or 100 μg/ml) or culture medium only (control) for 4 h before cathepsin B activity measurement by the Magic Red^®^ assay. Results are the mean ± SEM of *n* = 3–6 experiments. Statistical differences from control cells as determined by ANOVA followed by the Dunnett’s test. ***p < 0.001. **(D)** Effect of cathepsin B inhibition on CD-induced IL-1β secretion. The cells were pre-incubated with CA-074Me (10 μM) or culture medium only for 1.5 h and then exposed to CDs (25 or 100 μg/ml) or culture medium only for 24 h. Results are the mean ± SEM of *n* = 3–5 experiments. Statistical differences as determined by ANOVA followed the Sidak’s test. ***p < 0.001, when compared to cells without inhibitor pre-treatment and CD exposure. ^$^ *p* < 0.05, when compared to cells with inhibitor pre-treatment and without CD exposure. ^#^ *p* < 0.05 and ^###^ *p* < 0.001, when compared to cells without inhibitor pre-treatment and with CD exposure.

Recently, it has been suggested that lysosomal damage and cathepsins can be implicated in pyroptosis by triggering inflammasome activation (Wang et al., 2018). Therefore, we investigated the effect of CDs on lysosome integrity. To do so, macrophages were incubated with the NPs for 4 or 24 h, and lysosomal integrity and swelling were assessed. As shown on Figure 3A, CDs (3–100 μg/ml) decreased macrophage lysosome integrity in a concentration-dependent manner, with a statistically significant effect at concentrations of 12 (*p* < 0.001), 25 (*p* < 0.001), 50 μg/ml (*p* < 0.001) and 100 μg/ml (*p* < 0.001) and a EC50 of 13.11 μg/ml (CI95 9.39–18.49 μg/ml). This EC50 is consistent with the EC50 of 17.82 μg/ml observed in the MTT assay. This loss in lysosomal integrity resulted in lysosomal swelling as demonstrated in cells treated with 25 μg/ml CDs using the lysosomal marker LysoTracker^®^ Red DND-99 (Figure 3B). These data suggest that CDs induce lysosomal dysfunction. To investigate the role of cathepsins in macrophage pyroptosis induced by CDs, we assessed cathepsin B activity in cells exposed to the NPs, as well as the effect of cathepsin B inhibition on CD-induced inflammasome activation. As shown on Figure 3, macrophage exposure to CDs (25 and 100 μg/ml, for 4 h) resulted in a 2.3- and 2.9-fold increase in cathepsin B activity, respectively (Figure 3C, *p* < 0.001 for both CD doses), and cathepsin B inhibition by CA-074Me was associated with a significant decrease in IL-1β secretion (Figure 3D, *p* < 0.001 for 25 μg/ml and *p* < 0.05 for 100 μg/ml).

## Discussion

Interaction of engineered NPs with immune cells is an important issue considering the fundamental role of the immune system in the host defense and in numerous diseases. Accordingly, close attention has been paid to NP immunotoxicity in the past decades. However, the mechanisms and molecular pathways by which NPs affect the viability and function of immune cells remain unclear. In the present study, we investigated the toxicity mechanisms of engineered carbon NPs towards macrophages by focusing on pyroptosis, a pro-inflammatory form of programmed cell death. We demonstrated that cationic CDs trigger inflammasome-dependent pyroptosis in macrophages via lysosomal dysfunction.

Although CDs are rapidly expanding nanomaterials, their toxicological profile remains poorly understood owing to the large diversity of starting materials and synthesis protocols that can be used for manufacturing these NPs, thus leading to CDs with widely variable physicochemical characteristics. Studies exploring the interaction of CDs with immune cells are particularly scarce in the literature. Anionic CDs prepared from glycerol were shown to be cytotoxic to murine macrophages (RAW 264.7 cells) and to induce pro-inflammatory cytokine (MIP-1α, MIP-1β and MIP-2) production, but at relatively high concentrations (62.5–500 μg/ml) (Lategan et al., 2018). In a comparative study on three kinds of anionic CDs, all NPs were however non cytotoxic to RAW 264.7 cells at 1 mg/ml (Ayaz et al., 2020). Both cationic and anionic CDs (6.25–400 μg/ml) produced from bovine serum albumin and citric acid, respectively, were non cytotoxic to RAW 264.7 cells up to 400 μg/ml, but cationic CDs induced an increase in TNF-α and IL-6 secretion from 100 μg/ml (Usman et al., 2020). Similarly, CDs displaying a net positive charge were reported to be more toxic than negatively charged CDs towards cultured fibroblasts (Havrdova et al., 2016). On our side, by screening a large library of CDs, we previously reported that anionic CDs prepared from citric acid (3–200 μg/ml) are not cytotoxic towards THP-1-derived macrophages, in contrast to cationic CDs (Fan et al., 2019). Taken all together, these studies suggests that cationic CDs tend to be cytotoxic in contrast to anionic ones. Thus, we conducted the present study on cationic CDs only. Worth to note however, that we previously demonstrated that a cationic charge is not sufficient to confer toxicity to CDs and that the surface charge density rather than the absolute value of ζ-potential is a more relevant toxicity descriptor (Fan et al., 2019; Weiss et al., 2021b). Thus, we selected herein a cationic CD with a high charge density. We thus confirmed that cationic CDs are cytotoxic towards macrophages, at concentrations lower or equal to 50 μg/ml. This observation is in agreement with the literature on engineered NPs, showing that surface charge influences NP biocompatibility, with a positive ζ-potential being associated with toxicological risks, due to greater damaging effect of cationic NPs on cell and/or lysosomal membrane or greater protein corona formation that drives NP cell uptake (Mahmoudi et al., 2011; Luyts et al., 2013; Gatoo et al., 2014). In this respect, we show herein that cationic CDs are internalized by macrophages which is in agreement with our previous reports (Ronzani et al., 2019; Weiss et al., 2021b). We did not assess CD cell uptake mechanisms in the present work, but we previously reported that internalization of cationic CDs with a high charge density is the consequence of endocytosis through the clathrin- and caveolae-mediated pathways, but also to some extent, phagocytosis and some energy-independent process (Weiss et al., 2021b). These uptake mechanisms could take place in the present study. Whether CDs enter cells via receptor-mediated mechanisms, and particularly via receptors implicated in pyroptosis such as Toll-Like receptor, is however unknown and deserves investigation.

NPs have been shown to induce cell death by various mechanisms including autophagy, ferroptosis, apoptosis and necrosis (Stern et al., 2012; Mohammadinejad et al., 2019; Zheng et al., 2021). Besides, various kinds of NPs, including carbon nanotubes (Meunier et al., 2012; Svadlakova et al., 2020), silver (Murphy et al., 2016), polymer (Nandi et al., 2021) or silica (Sandberg et al., 2012; Peeters et al., 2014) NPs have been reported to activate NLRP3 inflammasome, suggesting that NPs could also trigger cell death by pyroptosis through the canonical pathway. In agreement with this hypothesis, involvement of inflammasome-dependent pyroptosis in the toxicity of several NPs in macrophages has been demonstrated (Reisetter et al., 2011; Mirshafiee et al., 2018; Wang et al., 2020). On their side, CDs have been reported to induce autophagy and apoptosis in cancer cells (Arkan et al., 2018; Bajpai et al., 2020; Jiao et al., 2020), but their capacity to trigger pyroptosis has not been investigated so far. In the present study, we provide evidence for the first time that inflammasome-dependent pyroptosis is involved in macrophage death induced by cationic CDs. Indeed, we found that: 1) caspase-1 is activated and IL-1β secretion is increased after cell exposure to CDs, 2) macrophage viability loss and inflammasome activation induced by CDs are associated with cell membrane leakage which is a hallmark of pyroptosis, 3) inhibition of caspase-1 significantly restores macrophage viability while limiting cell leakage. Pyroptosis can be activated through a non-canonical pathway that is mediated by caspase-4/5/11 activation (Hachim et al., 2020). As well, recently caspase-3 activation has been associated with death by pyroptosis in cancer cells (Yu et al., 2021). Whether CDs could induce pyroptosis through caspase-4/5/11 or caspase-3 activation remains unexplored yet.

A link has been established between inflammasome activation and lysosomal dysfunction, with a key role of lysosomal damage and protease cathepsin B release (Hornung et al., 2008; Svadlakova et al., 2020). Indeed, the lysosome is well known to sequester NPs after their cell uptake which can result in lysosomal dysfunction (Stern et al., 2012). In the present work, we found an increase in cathepsin B activity after macrophage exposure to CDs, and a decrease in CD-induced IL-1β secretion in the presence of a cathepsin B inhibitor, suggesting a role of lysosome in CD-induced inflammasome activation, and possibly macrophage death by pyroptosis. These data are in agreement with our previous observation that cationic CDs traffick to lysosome upon macrophage internalization and that lysosome plays a central role in the toxicological effects of the NPs (Ronzani et al., 2019). In the literature, several mechanisms of inflammasome activation in response to NPs have been proposed, among which increased ROS production was most frequently described (Hussain et al., 2009; Tschopp and Schroder, 2010; Zhang et al., 2018; Wang et al., 2019). While we did not measure oxidative stress in the present study, we and others showed that cationic CDs are capable of inducing ROS production (Havrdova et al., 2016; Ronzani et al., 2019). Therefore, activation of inflammasome and pyroptosis induced by CDs could result from cathepsin B release after lysosomal integrity loss, but also oxidative stress.

Due to its swelling nature, pyroptosis results in the release of pro-inflammatory cytokines such as IL-1β and other DAMPs, also known as alarmins (Cookson and Brennan, 2001; Vande Walle and Lamkanfi, 2016). These DAMPs help the body to fight foreign assaults by recruiting and/or activating immune and structural cells, to initiate or promote local or systemic inflammation. As inflammation constitutes a central pathological process in a myriad of diseases, pyroptosis is thought to participate to several disorders, including immune and cardiovascular diseases, as well as cancer (Ma et al., 2018). Macrophage pyroptosis in response to CDs could thus contribute to the *in vivo* toxicity of the nanomaterials, as demonstrated for silica NPs and cardiac hypertrophy or hepatotoxicity (Zhang et al., 2018; Wang et al., 2022). Besides, taking into account its pathological role, pyroptosis is considered as a potential therapeutic target in several diseases, particularly cancer (Wang et al., 2021). So far various strategies have been investigated to induce pyroptosis with the purpose of treating cancer. Among these strategies, nanomaterials appear attractive (Wu et al., 2021). Thus, cationic CDs as described herein could find therapeutic application in the field of cancer.

## Conclusion

**FIGURE 4:**
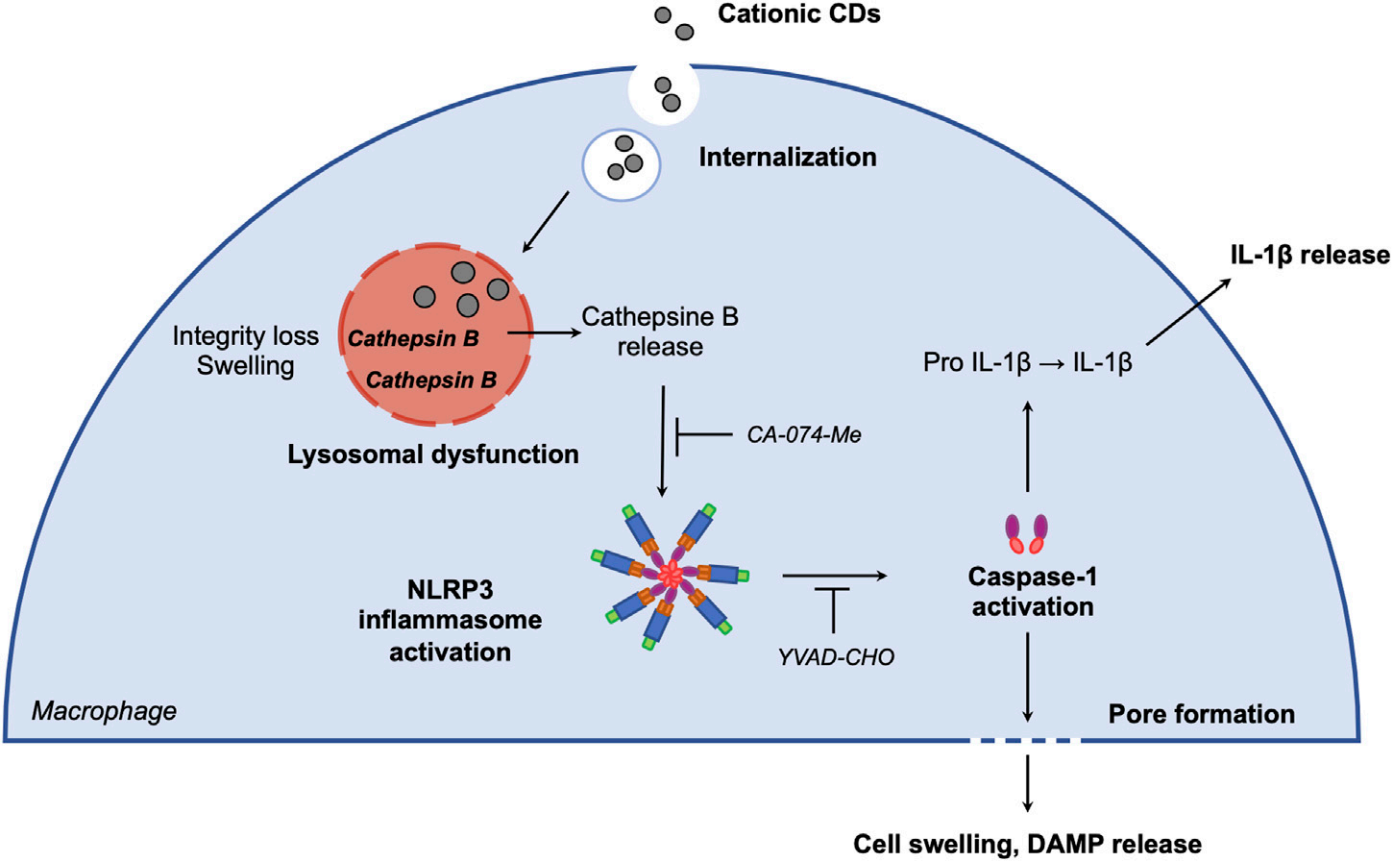
Proposed mechanisms of CD-induced macrophage pyroptosis. After macrophage internalization, CDs traffick to the lysosome. CD accumulation in the organelle alters membrane integrity and induces lysosome swelling leading to cathepsin B release. Cathepsin B in turn activates NLRP3 inflammasome, which induces IL-1β release and cell swelling through caspase-1 activation.

In summary, we demonstrate here that cationic carbon NPs with a high charge density induce inflammasome-dependent pyroptosis in macrophages *via* lysosomal dysfunction. The proposed mechanisms of CD-induced macrophage pyroptosis involves CD internalization and trafficking to the lysosome, resulting in lysosome membrane integrity loss and lysosome swelling leading to cathepsin B release. Cathepsin B in turn activates NLRP3 inflammasome, which induces IL-1β release and cell swelling through caspase-1 activation (Figure 4). These data provide new insights into interaction of carbon nanoparticles with the immune system, and particularly macrophages which play a central role in the host defense. They bring also useful information for the development of safe-by-design nanomaterials and the use of these nanomaterials as therapeutic tools in the treatment of cancer through pyroptosis induction.

## Data Availability

The raw data supporting the conclusion of this article will be made available by the authors, without undue reservation.
